# ASSOCIATION OF MID UPPER ARM CIRCUMFERENCE AND COGNITION: A POPULATION-BASED COHORT STUDY

**DOI:** 10.1093/geroni/igac059.327

**Published:** 2022-12-20

**Authors:** David Lynch, Annie Green Howard, Hsiao-Chuan Tien, Shufa Du, Bing Zhang, Huijun Wang, Penny Gordon-Larsen, John Batsis

**Affiliations:** UNC, Chapel Hill, North Carolina, United States; University of North Carolina at Chapel Hill, Chapel Hill, North Carolina, United States; UNC Chapel Hill, Chapel Hill, North Carolina, United States; University of North Carolina at Chapel Hill, Chapel Hill, North Carolina, United States; National Institute for Nutrition and Health Chinese Center for Disease Control and Prevention, China, Beijing, China (People's Republic); National Institute for Nutrition and Health Chinese Center for Disease Control and Prevention, Beijing, Beijing, China (People's Republic); University of North Carolina at Chapel Hill, Chapel Hill, North Carolina, United States; University of North Carolina at Chapel Hill, Chapel Hill, North Carolina, United States

## Abstract

**Introduction:**

Evidence suggests a positive association between muscle mass and cognitive impairment exists. Mid-upper arm muscle circumference (MUMC) is a simple measure that may provide prognostic information on cognitive status.

**Methods:**

We included adults aged ≥55 years from the China Health and Nutrition Survey 1997-2018 with MUAC and triceps skinfold (TSF) measurements at each visit. Cognition was estimated based on a subset of the modified Telephone Interview for Cognitive Status (TICS, 0─27). Sex-stratified linear mixed-effects models accounting for within-individual and within-community correlation assessed the association between MUAC and the ratio of MUAC:TSF with TICS across age. We tested whether the rate of cognitive decline by age differed by quartiles of MUAC and MUAC:TSF in separate models. In cases of no statistical differences in cognitive declines by age, we tested whether overall cognitive function was associated with quartiles of MUAC and MUAC:TSF across all ages.

**Results:**

Of 5,964 adults (53% female, age 62.4±6.4), mean MUAC was 26.6±3.74 and 26.2±3.9 cm, mean MUAC:TSF ratio was 2.9±1.6 and 1.94±1.1, and baseline TICS was 15.4±6.1 and 13.2±6.4 for men and women, respectively. MUAC was not associated with the rate of cognitive decline. Lower MUAC was associated with higher overall cognitive function scores for men (p=0.01) and women (p=0.05). For men and women there was no association between MUAC:TSF ratio and either cognitive decline or overall function.

**Conclusion:**

MUMC can be a marker to predict overall cognitive function across this period in the lifecycle, suggesting that declining MUAC may help predict lower overall cognitive function

